# Preoperative splenic area as a prognostic biomarker of early-stage non-small cell lung cancer

**DOI:** 10.1186/s40644-023-00640-0

**Published:** 2023-12-01

**Authors:** Mengmei Liu, Guanghong Yan, Yanli Li, Ruiming You, Lizhu Liu, Dafu Zhang, Guangjun Yang, Xingxiang Dong, Yingying Ding, Shan Yan, Dingyun You, Zhenhui Li

**Affiliations:** 1 Yunnan Provincial Key Laboratory of Public Health and Biosafety, Kunming Medical University, 1168 West Chunrong Road, Chenggong District, Kunming, 650500 Yunnan P. R. China; 2 Department of Radiology, Yunnan Cancer Hospital, The Third Affiliated Hospital of Kunming Medical University, Yunnan Cancer Center, Kunming, 650118 China; 3 Institute of Biomedical Engineering, Kunming Medical University, 1168 West Chunrong Road, Chenggong District, Kunming, 650500 Yunnan P. R. China

**Keywords:** NSCLC, Modality, CT, Overall survival, Splenic area

## Abstract

**Background:**

The correlation between the preoperative splenic area measured on CT scans and the overall survival (OS) of early-stage non-small cell lung cancer (NSCLC) patients remains unclear.

**Methods:**

A retrospective discovery cohort and validation cohort consisting of consecutive NSCLC patients who underwent resection and preoperative CT scans were created. The patients were divided into two groups based on the measurement of their preoperative splenic area: normal and abnormal. The Cox proportional hazard model was used to analyse the correlation between splenic area and OS.

**Results:**

The discovery and validation cohorts included 2532 patients (1374 (54.27%) males; median (IQR) age 59 (52–66) years) and 608 patients (403 (66.28%) males; age 69 (62–76) years), respectively. Patients with a normal splenic area had a 6% higher 5-year OS (n = 727 (80%)) than patients with an abnormal splenic area (n = 1805 (74%)) (*p* = 0.007) in the discovery cohort. A similar result was obtained in the validation cohort. In the univariable analysis, the OS hazard ratios (HRs) for the patients with abnormal splenic areas were 1.32 (95% confidence interval (CI): 1.08, 1.61) in the discovery cohort and 1.59 (95% CI: 1.01, 2.50) in the validation cohort. Multivariable analysis demonstrated that abnormal splenic area was independent of shorter OS in the discovery (HR: 1.32, 95% CI: 1.08, 1.63) and validation cohorts (HR: 1.84, 95% CI: 1.12, 3.02).

**Conclusion:**

Preoperative CT measurements of the splenic area serve as a prognostic indicator for early-stage NSCLC patients, offering a novel metric with potential implications for personalized therapeutic strategies in top-tier oncology research.

**Supplementary Information:**

The online version contains supplementary material available at 10.1186/s40644-023-00640-0.

## Background

Non-small cell lung cancer (NSCLC) remains the leading cause of cancer-related deaths worldwide [1, 2]. Despite surgery being the primary treatment for patients with early-stage NSCLC [3], approximately 25-50% of NSCLC patients experience local recurrence or death following major surgery [4, 5]. Identifying prognostic markers is crucial in the management of cancer, as they enable stratification of patients for treatment by identifying those with different outcome risks [6]. However, current issues with prognostic markers, particularly in cancers that exhibit tumor heterogeneity and patient response variability, make it challenging to identify reliable markers [7]. Additionally, some prognostic markers lack sufficient validation or have limited generalizability to diverse patient populations [8]. Using several reported prognostic markers in NSCLC patients may lead to increased costs [9] or potential harm [10, 11]. Therefore, identifying new, inexpensive, and noninvasive biomarkers is essential for identifying individuals with a high mortality risk and initiating early interventions to delay disease progression.

Recent studies have highlighted the important role of the immune system in cancer development and treatment, as evidenced by the ability of specific immune cells to attack cancer cells and the effectiveness of immune-boosting therapies [12, 13]. New diagnostic and prognostic tools based on immune markers may guide treatment decisions and improve lung cancer patient outcomes [14]. The spleen, a unique organ in its cellular makeup and physical structure, contains numerous immune cells, including lymphocytes and macrophages, which are vital components of the body’s immune system [15]. Recent evidence suggests that splenomegaly, low preoperative splenic density, and large splenic volume (SV) may have a negative impact on the prognosis of patients with various cancer types who have received immunotherapy [16–19]. However, the area of the spleen and its relationship with the prognosis of patients with early-stage NSCLC have not been studied.

In this study, we aimed to investigate the association between the preoperative splenic area (splenic area) and the outcome of early-stage NSCLC patients. We hypothesized that the preoperative splenic area would be a prognostic biomarker of early-stage NSCLC.

## Methods

### Ethics approval and informed consent

The retrospective study was approved by the ethical council of the cancer hospital, and the requirement for informed consent was waived as the study was retrospective. To ensure confidentiality, all patient data collected from the survey were made anonymous. This study adhered to the guidelines provided by STROBE (Strengthening the Reporting of Observational Studies in Epidemiology) to ensure transparent and comprehensive reporting of the study’s findings [20].

### Patients

A total of 3172 patients were obtained through retrospective data collection and formed two cohorts: the discovery and the validation cohorts.

The discovery cohort included all consecutive non-small cell lung cancer patients who had undergone a chest or abdomen CT scan preoperatively and radical lung cancer resection in a comprehensive cancer center from January 2012 to December 2018. Patients with stage IV disease, splenectomy, lack of reliable CT scans, poor CT image quality or no available survival data were excluded. Poor image quality refers to the visual appearance of an image that is below the expected or desired level of clarity, sharpness, and overall visual fidelity.

After evaluating various databases based on factors such as the size and diversity of the dataset, the quality and completeness of the data, and the inclusion of complete preoperative CT images with survival time and status, we selected two relevant databases in the validation cohort. A total of 608 patients were obtained from the TCIA database (https://wiki.cancerimagingarchive.net) [21]. Patients with splenectomy, a lack of CT images, or poor CT image quality were excluded. Finally, 633 NSCLC patients from two medical centers in the United States (validation cohort A, n = 211) [22] and NSCLC patients who were treated at the MAASTRO Clinic (validation cohort B, n = 422) were included [23]. Validation cohort A was previously reported in the Journal of Biochem Pharmacol, while data for validation cohort B were referred to the Lung1 dataset in the Journal of Nature Communications [24, 25].

### Calculation of the splenic area

All patients’ preoperative CT scans were examined. First, using 3D Slicer (version 5.2.1) (https://www.slicer.org), the radiologist manually segmented the spleen on the slice that was located at the level of the splenic hilus on unenhanced or enhanced CT imaging. Second, after segmentation, the splenic area was measured (Supplemental Fig. 1). One abdominal radiologist with 13 years of experience and another with 2 years of experience who were blinded to the clinical data independently measured the splenic area. When the correlation coefficient between the measurements of these two radiologists was less than 0.90, a third radiologist with more than 25 years of experience measured the splenic area again, and the measurement result was the final result. Otherwise, the final result was the mean of the two measurements.

### Outcome

The primary outcome of this study was overall survival (OS), which was calculated as the time from surgery to death or the last follow-up. The data of patients who died or were lost to follow-up were reviewed. All eligible patients were followed up through electronic medical records and telephone. The final date of follow-up was October 13, 2022.

### Covariates

The analysis included the following covariates: age, sex, preoperative carcinoembryonic antigen (CEA) level (ng/ml), smoking history (never smoker, current or former smoker), tumor location, surgical approach (thoracoscopy or thoracotomy), tumor differentiation, T stage (T1-T4), N stage (N0-N3), pathology stage (I-III), histologic type, pleural invasion, and adjuvant chemotherapy. These covariates were included in the analysis as they were considered to potentially affect the outcome of interest, and their incorporation was necessary to account for their potential confounding effects.

### Statistical analysis

Prior to conducting data analysis, we assessed the predictor variables in the cohorts for missing values. Any data that were missing were excluded from the analysis to ensure eligibility. For continuous variables with normal distributions, we defined means and standard deviations (SD) and performed independent two-sample t tests. For categorical variables, we analysed the number and percentage of patients and used chi-square (χ^2^) tests.

To relax the assumption of a linear relationship between continuous predictors and death risks, we categorized the splenic area using restricted cubic splines (RCS) [26]. Previous research has established links between splenic size and volume and sex, age, and weight [27]. We identified optimal cut-off values for men and women separately based on the curve-fitting results. For men, the cut-off value was the lowest point of the curve, and the splenic area within the upper 25% and lower 75% of the inflection points was classified as the abnormal group, while the remainder was considered the normal group. For women, the cut-off value was also the lowest point of the curve, and the splenic area within the upper 67% and lower 33% of the inflection point was classified as the abnormal group, while the remainder was considered the normal group (Supplement Table 1).

**Table 1:** Characteristics of the cohort at baseline in the discovery cohort

Variable	Total (n = 2532)^1^	Splenic area		*P* value^2^
		Normal (n = 727)^**1**^	Abnormal (n = 1805)^**1**^	
Age, years				
Mean (SD)	58.82 (9.56)	58.24 (9.33)	59.05 (9.64)	0.05
Median (IQR)	59.00 (52.00,66.00)	57.00 (52.00,65.00)	59.00 (52.00,66.00)	0.04
Sex, n (%)				<0.001
Female	1158 (45.73)	383 (52.68)	775 (42.94)	
Male	1374 (54.27)	344 (47.32)	1030 (57.06)	
Smoking history, n (%)				0.008
Never smoker	1476 (58.29)	457 (62.86)	1019 (56.45)	
Current or former smoker	1022 (40.36)	259 (35.63)	763 (42.27)	
Unknown	34 (1.34)	11 (1.51)	23 (1.27)	
Preoperative CEA, ng/mL				
Mean (SD)	13.82 (156.60)	12.14 (85.85)	14.50 (177.35)	0.67
Median (IQR)	3.30 (2.05,5.79)	3.11 (2.04,5.70)	3.34 (2.05,5.80)	0.23
Tumor location, n (%)				0.61
Upper lobe	1277 (50.43)	356 (48.97)	921 (51.02)	
Non–upper lobe	1248 (49.29)	369 (50.76)	879 (48.70)	
Unknown	7 (0.28)	2 (0.28)	5 (0.28)	
Surgical approach, n (%)				0.63
Thoracoscope	1591 (62.84)	457 (62.86)	1134 (62.83)	
Thoracotomy	837 (33.06)	236 (32.46)	601 (33.30)	
Unknown	104 (4.11)	34 (4.68)	70 (3.88)	
Tumor differentiation, n (%)				0.045
Moderate-Well	288 (11.37)	69 (9.49)	219 (12.13)	
Poor-undifferentiated	126 (4.98)	29 (3.99)	97 (5.37)	
Other	2118 (83.65)	629 (86.52)	1489 (82.49)	
T stage, n (%)				0.80
T1	1089 (43.01)	305 (41.95)	784 (43.43)	
T2	962 (37.99)	275 (37.83)	687 (38.06)	
T3	293 (11.57)	93 (12.79)	200 (11.08)	
T4	184 (7.27)	53 (7.29)	131 (7.26)	
Unknown	4 (0.16)	1 (0.14)	3 (0.17)	
N stage, n (%)				0.43
N0	1546 (61.06)	453 (62.31)	1093 (60.55)	
N1	233 (9.20)	68 (9.35)	165 (9.14)	
N2-N3	350 (13.82)	104 (14.31)	246 (13.63)	
Unknown	403 (15.92)	102 (14.03)	301 (16.68)	
Histologic Stage, n (%)				0.15
I	1216 (54.63)	348 (53.95)	868 (54.90)	
II	410 (18.42)	133 (20.62)	277 (17.52)	
III	526 (23.63)	151 (23.41)	375 (23.72)	
Unknown	380 (15.01)	95 (13.07)	285 (15.79)	
Histologic type, n (%)				0.71
Adenocarcinoma	1961 (77.45)	569 (78.27)	1392 (77.12)	
Squamous cell carcinoma	454 (17.93)	128 (17.61)	326 (18.06)	
Other	117 (4.62)	30 (4.13)	87 (4.82)	
Pleural invasion, n (%)				0.66
Yes	425 (16.79)	121 (16.64)	304 (16.84)	
No	2064 (81.52)	591 (81.29)	1473 (81.61)	
Unknown	43 (1.70)	15 (2.06)	28 (1.55)	
Adjuvant chemotherapy, n (%)				0.76
Yes	1216 (48.03)	357 (49.11)	859 (47.59)	
No	1075 (42.46)	304 (41.82)	771 (42.71)	
Unknown	241 (9.52)	66 (9.08)	175 (9.70)	

**Note**:

^**1**^ Data are median (IQR)/ Mean (SD) or n (%)

^**2**^*P*-value, using Wilcoxon Mann-Whitney test, chi-square test or exact Fisher test depending on whether the variable is continuous or categorical

We calculated the 5-year OS using Kaplan‒Meier curves and compared the differences between the groups using the log-rank test. Hazard ratios (HRs) with two-sided 95% confidence intervals (CIs) were obtained for each group using Cox proportional risk regression models. We used multivariate Cox proportional risk regression analysis to identify independent risk factors for death and select variables that may affect prognosis. We used two models: model 1 adjusted for age, and model 2 further adjusted for smoking history, tumor location, tumor differentiation, histologic stage, histologic type, pleural invasion, and adjuvant chemotherapy. Subgroup analysis was conducted after stratification by age, sex, tumor site, surgical approach, pathology stage, histologic type, pleural invasion, and adjuvant treatment to investigate potential causes of heterogeneity. For missing value processing in classification variables, we set them as dummy variables. We used the R package “Forestplot” to generate forest plots for subgroup stratification analysis. Statistical significance was determined using a p value of less than 0.05. Data processing and analysis were performed using R software (version 4.2.1).

## Results

### Clinicopathologic characteristics

#### Discovery cohort

**Fig. 1:**
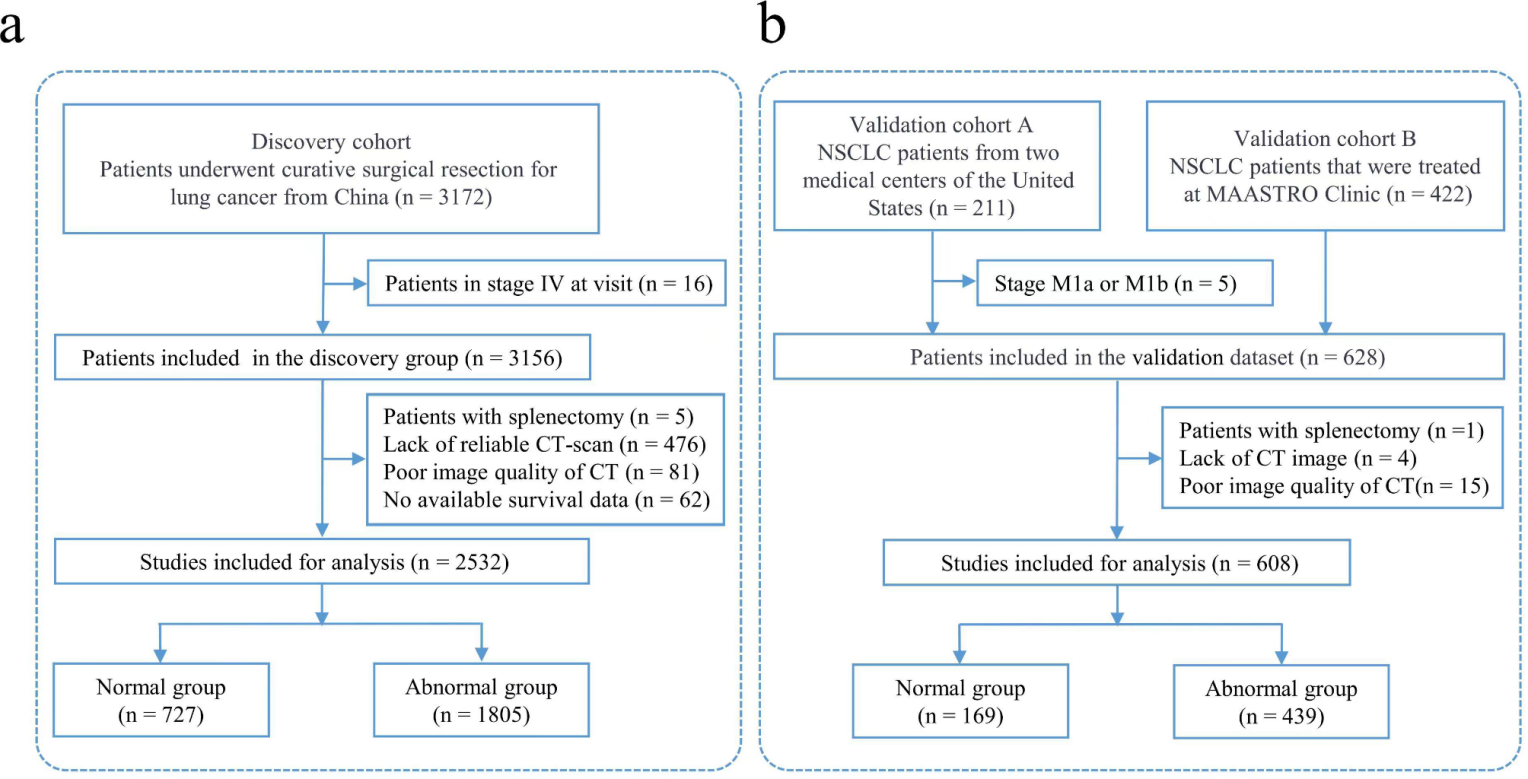
Patient study follow chart. Discovery cohort **(a)**; Validation cohort **(b)**

The preliminary analysis included 2532 patients in total. Figure 1a shows the number of participants who were evaluated for eligibility as well as the reasons for exclusion. Normal (n = 727) and abnormal (n = 1805) patients were studied, with 1374 (54.27%) males and 1158 (45.73%) females. The mean (SD) age in the total population and the normal and abnormal groups was 58.82 (9.56), 58.24 (9.33), and 59.05 (9.64), respectively. The median and interquartile range (IQR) of the splenic area in the total population and the normal and abnormal groups were 27.37 (22.03–33.74) cm^2^, 25.37 (20.76–30.83) cm^2^ and 29.09 (23.43–35.70) cm^2, respectively^. Thoracoscopic surgery was performed in 1591 (62.84%) patients, and thoracotomy was performed in 837 (33.06%) patients. A total of 1216 (48.03%) patients received adjuvant chemotherapy. The characteristics of the discovery cohort are shown in Table 1.

#### Validation cohort

A total of 608 patients were involved in the study. Validation cohort A (NSCLC patients from two medical centers in the United States, n = 211) and validation cohort B (NSCLC patients treated at the MAASTRO Clinic, n = 422) were among them. The number of participants who were assessed for eligibility and the reasons for exclusion are depicted in Fig. 1b. Normal (n = 169) and abnormal (n = 439) patients were included, with 205 (33.72%) males and 403 (66.28%) females. The mean (SD) age in the total population and the normal and abnormal groups was 68.12 (9.99), 68.29 (9.62), and 68.05 (10.14) years, respectively. The median (IQR) of the splenic area in the total population and the normal and abnormal groups were 33.28 (26.78–425) cm^2^, 29.76 (23.65–35.12) cm^2^ and 35.51 (28.40–43.88) cm^2^, respectively. A total of 41 (48.03%) patients received adjuvant chemotherapy. Supplemental Table 2 shows the characteristics of the validation cohort.

### Comparison of clinical characteristics of discovery and validation cohorts

The discovery and validation cohorts differed significantly in both demographic and pathological characteristics (all *p* < 0.05). Among them, the splenic area (median (IQR)) in the validation cohort was significantly larger than that in the discovery cohort of patients (33.28 (26.78–425) cm^2^ vs. 27.37 (22.03–33.74) cm^2^, *p* < 0.001) (Supplement Table 3).

### The relationship between the splenic area and 5-year OS

#### Discovery cohort

**Fig. 2:**
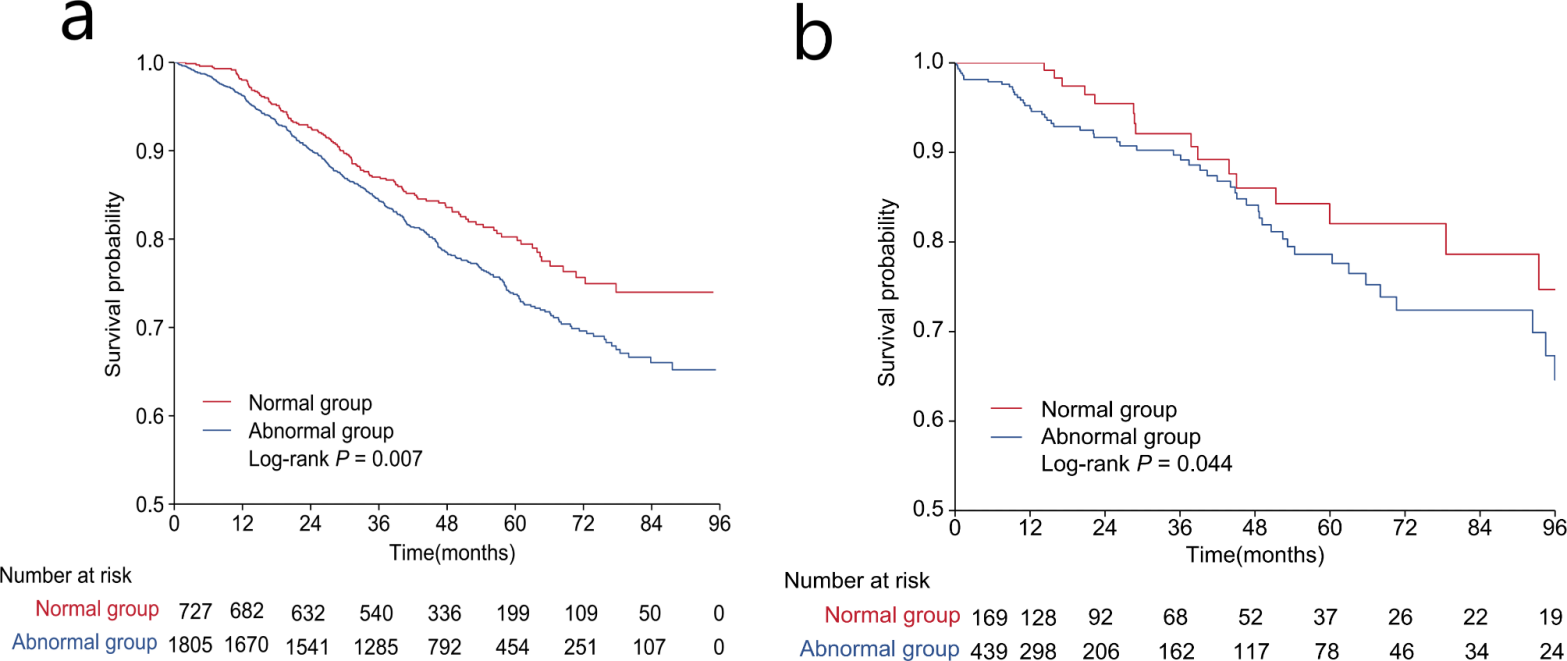
Survival analysis comparing patients in the normal group and patients in the abnormal group. Total population of the discovery cohort **(a)**; total population of the validation cohort **(b)**

The cut-off values of the splenic area in the abnormal group were 29.37 to 36.05 cm^2^ for men and 25.50 to 33.85 cm^2^ for women based on the curves (Supplement Table 1). Supplemental Fig. 2a, b, and c demonstrate the U-shaped linear relationship between the unadjusted risk ratio of death and splenic area in the total population and the male and female subgroups, respectively. Figure 2a shows the overall survival rates of the normal and abnormal splenic area groups. The difference in 5-year OS between patients with normal and abnormal splenic areas was significantly different (80% (77%, 84%) vs. 74% (71%, 76%); log-rank *p* = 0.007). In univariate analysis, the abnormal splenic area group in the total population was associated with decreased OS (HR 1.32, 95% CI: (1.08,1.61), *p* = 0.007). Univariate analysis by sex obtained the same result in males (HR 1.30, 95% CI: (1.01, 1.67), *p* = 0.045) but no significant difference in females (HR 1.18, 95% CI: (0.86, 1.63), *p* = 0.31) (Table 2). The multivariate analysis results were consistent with the univariate analysis, with the splenic area as a binary dependent variable in the total population remaining an independent poor prognostic factor for OS (model 1: HR: 1.29, 95% CI: (1.06,1.58), *p* = 0.01; model 2: HR: 1.32, 95% CI: (1.08,1.63), *p* = 0.008) (Table 2).

**Table 2:** Univariate and multivariate-adjusted association of splenic area and overall survival in the discovery and validation cohort

Variable	Univariate analysis			Multivariate analysis (M1)^1^			Multivariate analysis (M2)^2^	
	HR (95% CI)	*P* value		HR (95% CI)	*P* value		HR (95% CI)	*P* value
**Discovery cohort**								
All patients								
Normal	Ref			Ref			Ref	
Abnormal	1.32(1.08,1.61)	0.007		1.29(1.06,1.58)	0.01		1.32(1.08,1.63)	0.008
Male								
Normal	Ref			Ref			Ref	
Abnormal	1.30 (1.01,1.67)	0.045		1.29 (1.00,1.67)	0.048		1.47 (1.13,1.93)	0.004
Female								
Normal	Ref			Ref			Ref	
Abnormal	1.18 (0.86,1.63)	0.31		1.17 (0.85,1.61)	0.35		1.07 (0.77,1.50)	0.69
**Validation cohort**								
All patients								
Normal	Ref			Ref			Ref	
Abnormal	1.59 (1.01,2.50)	0.045		1.79 (1.12,2.86)	0.02		1.84 (1.12,3.02)	0.02
Male								
Normal	Ref			Ref			Ref	
Abnormal	1.54 (0.88–2.68)	0.13		1.5 (0.86,2.62)	0.15		1.57 (0.86,2.86)	0.14
Female								
Normal	Ref			Ref			Ref	
Abnormal	1.67 (0.75,3.74)	0.21		3.67(1.34,10.04)	0.01		3.83 (1.12,13.18)	0.03

**Note**:

^**1**^Multivariate analysis (M1) was adjusted for age (continuous)

^**2**^Multivariate analysis (M2) was adjusted for multivariate analysis (M1) plus smoking history, preoperative CEA, tumor location, surgical approach, tumor differentiation, histologic stage and histologic type, pleural invasion and adjuvant chemotherapy

#### Validation cohort

The cut-off values of the splenic area were 23.06 to 314 cm^2^ for males and 26.12 to 33.86 cm^2^ for females in the abnormal group (Supplement Table 1). Supplemental Fig. 2d, e, and f demonstrate the U-shaped linear relationship between the unadjusted risk ratio of death and splenic area in the total population and in the male and female subgroups, respectively. Figure 2b shows the overall survival rates of the normal and abnormal splenic area groups at different times. Among them, the difference in 5-year OS between patients with normal and abnormal splenic areas was significantly different (82% (73%, 92%) vs. 79% (73%, 85%); log-rank *p* = 0.044). In the univariate analysis, the abnormal splenic area group in the total population was associated with decreased OS (HR: 1.59, 95% CI: (1.01,2.50), *p* = 0.045). Univariate analysis by gender showed no significant difference in either men or women (HR 1.54, 95CI: (0.88,2.68), *p* = 0.13; HR1. 67, 95CI: (0.75,3.74), *p* = 0.21) (Table 2). In the adjusted multivariate analysis, splenic area as a binary dependent variable in the total population remained an independent poor prognostic factor for OS (model 1 h: 1.79, 95CI: (1.12,2.68), *p* = 0.02; model 2 HR 1.84, 95CI: (1.12,3.02), *p* = 0.02) (Table 2).

#### Subgroup analysis

**Fig. 3:**
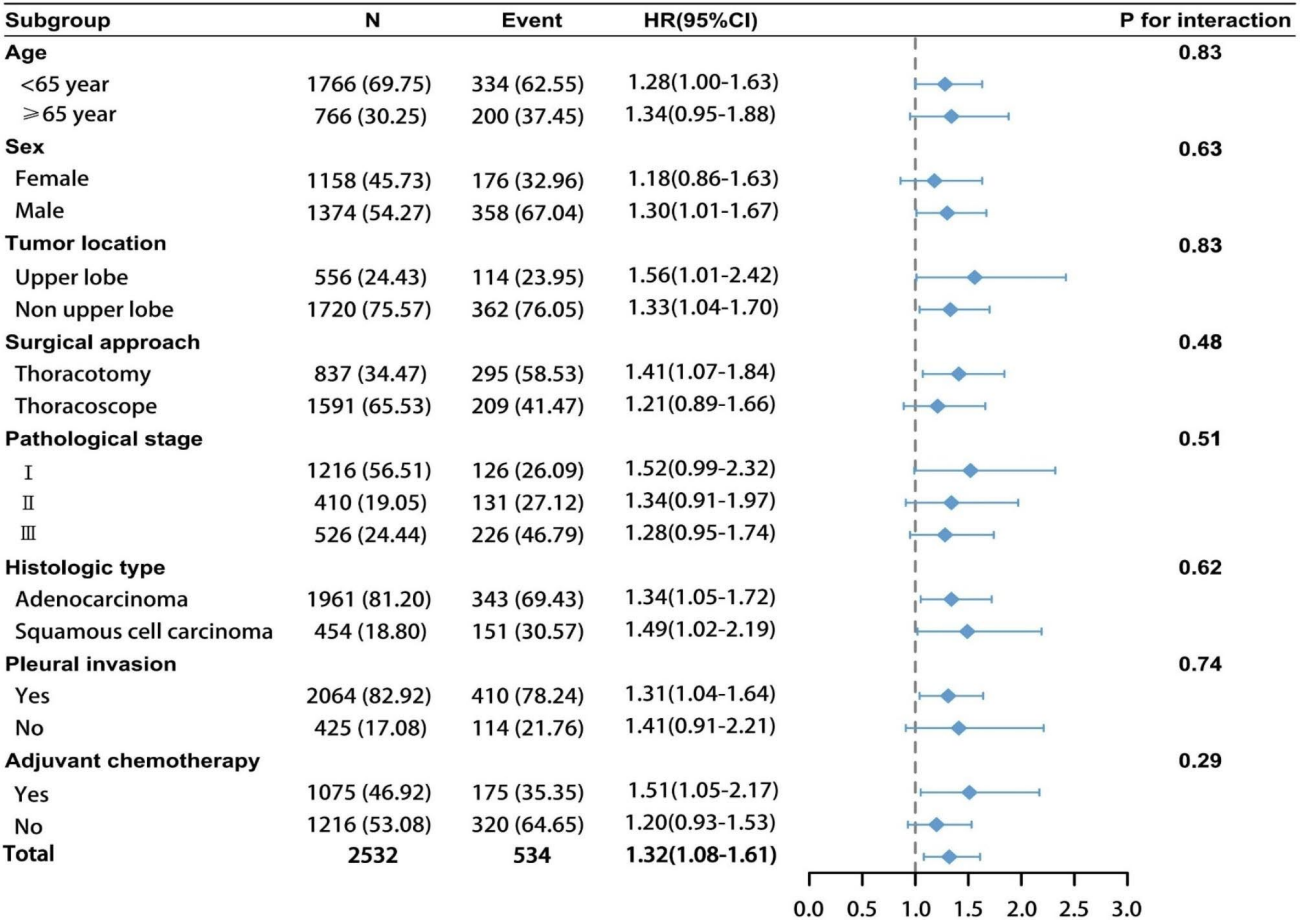
Forest plot of the abnormal group stratified by clinicopathological variables in the discovery cohort

Patients with abnormal splenic areas had a higher HR in most subgroups, similar to the general population (Fig. 3). The baseline characteristics of the patients and the abnormal area of the spleen did not interact significantly (all *p* > 0.05).

## Discussion

Our study found that the preoperative splenic area measured by CT can serve as an independent predictor of OS in early-stage NSCLC patients, as confirmed by publicly available data. These results suggest that the splenic area reflects cancer-related mechanisms and may merit being integrated into lung cancer treatment algorithms. In this study, we included patients independent of stage and histological subtypes as a reflection of the inhomogeneous lung cancer population. By doing so, we could test the true value of this potential prognostic marker in an everyday clinical setting.

Previous studies have established a potential link between splenic volume and treatment response in NSCLC patients receiving immunotherapy [16–19]. For example, in metastatic NSCLC, where systemic therapies such as immunotherapy are predominant, the evidence suggesting splenic volume as a surrogate marker for the efficacy of immune checkpoint inhibitors is compelling [18]. This correlation might be attributed to the spleen’s integral role in immune modulation and response to systemic therapies. However, translating these findings to a surgical context presents challenges due to the distinct nature of the treatment and disease progression. Additionally, the intriguing findings from studies on patients with sepsis, where preoperative splenic volume was shown to be an independent predictor of OS, add another layer of complexity [28]. These studies highlight the potential of spleen metrics as a prognostic tool, yet their applicability to NSCLC patients undergoing surgery remains to be explored. In this uncharted territory, our study makes a novel contribution by evaluating the prognostic value of the preoperative splenic area, measured by CT, in early-stage NSCLC patients who had undergone radical lung cancer resection. This focus on a surgical cohort is crucial, as it may uncover different dynamics in the prognostic value of spleen metrics compared to those observed in patients undergoing immunotherapy. Our findings suggest that the preoperative splenic area is indeed a significant predictor of overall survival in early-stage NSCLC patients. This observation not only adds a new dimension to the literature on splenic metrics in cancer but also raises questions about the underlying mechanisms that might govern this relationship in the context of surgical treatment for NSCLC. In light of these insights, future research should aim to unravel the complex interactions between splenic metrics and cancer outcomes in various treatment modalities, including surgery. Such studies will be instrumental in refining prognostic tools and potentially tailoring treatment strategies based on spleen metrics in NSCLC patients.

A study retrospectively investigated 232 patients with sepsis [28] and showed that the splenic volume of patients with sepsis appeared to be associated with mortality. They found that patients with a smaller splenic volume had a significantly higher risk of mortality than those with a normal splenic volume. However, we found a U-shaped association between splenic area and mortality. That is, patients in larger and smaller splenic areas both had worse outcomes. We did not perform stratified analysis in the abnormal splenic area group due to numerical limitations but confirmed our findings in subgroup analyses of different pathological stages, tissue types, etc. More prospective and multicenter cohort studies with large samples are needed to confirm the “U” association between the splenic area and prognosis of NSCLC.

Most of the studies investigated the relationship between the spleen and cancer outcome based on splenic volume [16–19]. The reason why we used the splenic area as a prognostic biomarker has important strengths. First, the splenic area is based on the largest cross-sectional area of the spleen and is independent of the shape or location of the spleen, thus reflecting the size of the splenic parenchyma more stably and consistently. In volumetric analysis, several factors can introduce errors and variability. The shape of the spleen, its position within the body, potential rotation, and deformation all contribute to challenges in accurately determining the volume. These factors can lead to inconsistencies in volumetric measurements across different patients or even in the same patient over time. In contrast, the splenic area is measured on a single axial CT slice that displays the spleen’s largest cross-sectional area. This approach minimizes the influence of shape and positional variations. By focusing on the maximum cross-sectional area, we reduce the potential for measurement variability that can arise from the aforementioned factors. The result is a more consistent and reproducible measurement, which is crucial in a clinical research setting. Additionally, splenic area measurement is simpler and faster. It only requires selecting one slice on CT, instead of measuring and summing multiple slices.

Possible explanations for these results are as follows. First, multiple studies have demonstrated a connection between certain types of cancer and chronic inflammation. This inflammation may weaken the immune system and help cancer grow by causing immunological inversion. It may be caused by aberrant myelopoiesis, a disease that causes myeloid-derived suppressor cells (MDSCs) to accumulate in the body [29, 30]. Similar to neutrophils, these cells are populations of immature myeloid cells that circulate in cancer patients. They suppress immune responses that are made against cancer. MDSCs inhibit the immune response by increasing tumor cell survival, invasion into healthy tissue, angiogenesis, and metastasis to promote cancer [31–35]. Second, in animal models, splenomegaly (enlarged spleen) is connected with MDSC accumulation. This association with hepatocellular cancer was discovered [36]. Third, in mouse models of lung cancer, the spleen is also a source of increased splenic myeloid progenitor cells, which can significantly boost the host response and affect tumor progression [37]. Finally, splenectomy increases liver metastases in colorectal cancer mouse models, highlighting the importance of the spleen in activating an immune response against the tumor [37]. This could be the cause of the worsening prognosis for NSCLC patients as the area of the spleen increases and decreases.

The findings of our study also support the idea that sex-related differences in spleen size may impact immune function and tumor malignancy in NSCLC patients. We found that male patients had a significantly higher splenic area than female patients, which is consistent with previous literature [38]. One factor that may contribute to the sex-related differences in spleen size is body size and weight [39]. Male patients are generally larger and heavier than female patients, which may result in a larger spleen size. In addition, hormones such as testosterone, which are present in higher levels in male patients, can stimulate red blood cell production, which can also lead to an increase in splenic size [40]. Our study also showed that the preoperative splenic area in the abnormal group was associated with significantly higher mortality in males (HR, 2.73; 95% CI, 54–4.82), although it was not a risk factor in females. This may be due to differences in the immune response between male and female patients. Specifically, studies have shown that female patients generally have a stronger immune response than male patients, which may play a role in their ability to fight off cancer cells [41]. This suggests that the splenic area may be a more useful prognostic marker in male NSCLC patients than in female patients. More research is needed to fully understand the relationship between sex-related differences in spleen size, immune function, and tumor malignancy in NSCLC patients.

One of the implications of our study is that the preoperative splenic area could serve as a simple, inexpensive, and noninvasive prognostic biomarker that could help screen high-risk patients with early-stage NSCLC and guide surgical treatment decisions. However, it is important to note that optimal cut-off values for the splenic areas depend on the sex of the patients in my study, as shown by our curve-fitting analysis. To effectively apply these findings in another clinical setting, we suggest that further research is warranted to validate our findings across different patient populations and healthcare settings. Such research could explore the application of preoperative splenic area measurements in various clinical scenarios, including their potential role in monitoring disease progression and response to treatment. Additionally, it would be beneficial to investigate the integration of this marker into existing clinical workflows and decision-making processes. This could involve developing guidelines for the interpretation of splenic area measurements, as well as training programs for radiologists and clinicians to enhance their understanding and utilization of this tool. Moreover, future studies should consider the interplay of the splenic area with other clinical parameters and biomarkers to develop a more comprehensive prognostic model for early-stage NSCLC. This holistic approach could lead to more personalized and effective treatment strategies, ultimately improving patient outcomes.

The large population and externally validated results of this study ensure the robustness of the findings under multiple conditions and the significant benefits of our study. However, one limitation is that due to the small number of patients with large and small spleens in our cohort, we cannot know whether large or small splenic areas are independently associated with poor prognosis in NSCLC patients. Second, we did not perform a randomized exposure analysis, so retrospective studies may suffer from issues such as confounding and selection bias. Therefore, prospective multicenter randomized controlled trials are needed to verify our results. Third, we did not collect immune parameters associated with splenic size and postoperative splenic area, so we could not determine the correlation between splenic area and related immune parameters or whether changes in splenic area would affect patient outcomes. Fourth, several major survival factors, such as surgeon experience and patient functional status, were not included in the data. These factors may have an impact on patients’ postsurgery survival times. Furthermore, we did not adjust for potential confounding factors that may affect the splenic area or NSCLC prognosis, such as comorbidities, medications, and lifestyle factors. Moreover, there was no information on the incidence and cause of death, as well as cancer specificity, recurrence-free survival, and disease-free survival. Finally, we did not compare the splenic area with other radiologic markers of spleen size or function, such as splenic density or perfusion, which may also have prognostic value. Therefore, our results should be interpreted with caution, and further prospective multicenter randomized controlled trials are needed to confirm our findings and elucidate the underlying mechanisms.

## Conclusion

Our study provides evidence that the preoperative splenic area may be a novel, simple, noninvasive, and repeatable radiologic marker that can be used for prognostic assessment and personalized treatment of patients with early-stage NSCLC. By continuing to explore and refine its application, we can improve the accuracy and personalization of prognostic assessment and provide guidance for treatment decisions.

### Electronic supplementary material

Below is the link to the electronic supplementary material.

Supplementary Material 1: Calculation of the splenic area: After definition of the patients cross-sectionally (panel 1), we used a signal intensity-based threshold approach (1 to 100 HU) to identify the area of the spleen (red, panel 2), a 57-year-old female in the abnormal group; splenic area: 747 cm^2^ **(a)**, a 67-year-old female in the abnormal group; splenic area: 16.3 cm^2^ **(b)**, a 77-year-old female in the normal group; splenic area: 30.01 cm^2^ **(c)**.

Supplementary Material 2: Mortality according to splenic area. Unadjusted (red line) hazards for mortality according to splenic area (cm^2^). Dashed lines represent 95% confidence intervals. The blue lines represent the fraction of the population with different levels of splenic area; total population of the discovery cohort **(a)**; male of the discovery cohort **(b)**; female of the discovery cohort **(c)**; total population of the validation cohort **(d)**; male of the validation cohort **(e)**; female of the validation cohort **(f)**.

Supplementary Material 3

## Data Availability

The validation cohort A and B datasets generated and/or analysed during the current study are available in the [The Cancer Imaging Archive (TCIA)] repository, [https://wiki.cancerimagingarchive.net]. Publication reference number for validation cohort A [Gevaert, O., Xu, J., Hoang, C. D., Leung, A. N., Xu, Y., Quon, A., … Plevritis, S. K. (2012, August). Non–Small Cell Lung Cancer: Identifying Prognostic Imaging Biomarkers by Leveraging Public Gene Expression Microarray Data—Methods and Preliminary Results. Radiology. Radiological Society of North America (RSNA). 10.1148/radiol.12111607]; validation cohort B [Aerts, H. J. W. L., Velazquez, E. R., Leijenaar, R. T. H., Parmar, C., Grossmann, P., Carvalho, S., Bussink, J., Monshouwer, R., Haibe-Kains, B., Rietveld, D., Hoebers, F., Rietbergen, M. M., Leemans, C. R., Dekker, A., Quackenbush, J., Gillies, R. J., Lambin, P. (2014, June 3). Decoding tumour phenotype by noninvasive imaging using a quantitative radiomics approach. Nature Communications. Nature Publishing Group. 10.1038/ncomms5006]. The development cohort datasets generated and/or analysed during the current study are not publicly available [Data containing personal or sensitive information] but are available from the corresponding author [Zhenhui Li, MD & PhD, Department of Radiology, the Third Affiliated Hospital of Kunming Medical University, Yunnan Cancer Hospital, Yunnan Cancer Center, Kunming, 650118, China. Tel: +86-871-68179549; E-mail: lizhenhui@kmmu.edu.cn] on reasonable request.

## Abbreviations

OS: Overall Survival
NSCLC: Non-small Cell Lung Cancer
CT: Computed tomography
HR: Hazard Ratio
IQR: Interquartile Range
CI: Confidence Interval
CEA: Carcinoembryonic Antigen
