# Supplementary figures and images for: Preoperative splenic area as a prognostic biomarker of early-stage non-small cell lung cancer
Source: Cancer Imaging. 2023 Dec 1;23:116. doi: 10.1186/s40644-023-00640-0 (PMC10691021; doi:10.1186/s40644-023-00640-0)

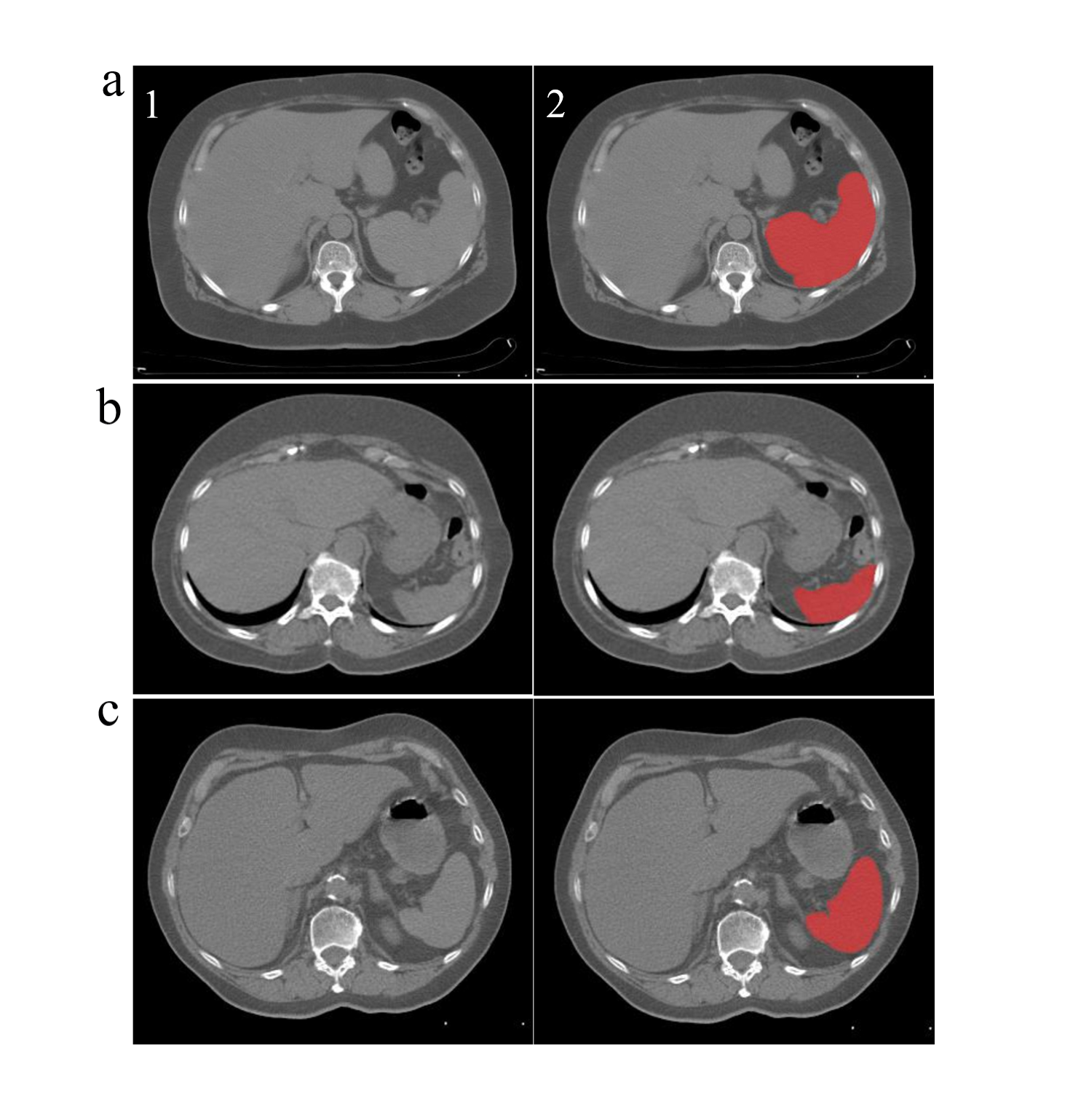

Supplement: Supplementary file 1 — Supplementary Material 1: Calculation of the splenic area: After definition of the patients cross-sectionally (panel 1), we used a signal intensity-based threshold approach (1 to 100 HU) to identify the area of the spleen (red, panel 2), a 57-year-old female in the abnormal group; splenic area: 747 cm2 (a), a 67-year-old female in the abnormal group; splenic area: 16.3 cm2 (b), a 77-year-old female in the normal group; splenic area: 30.01 cm2 (c). [file 40644_2023_640_MOESM1_ESM.png]

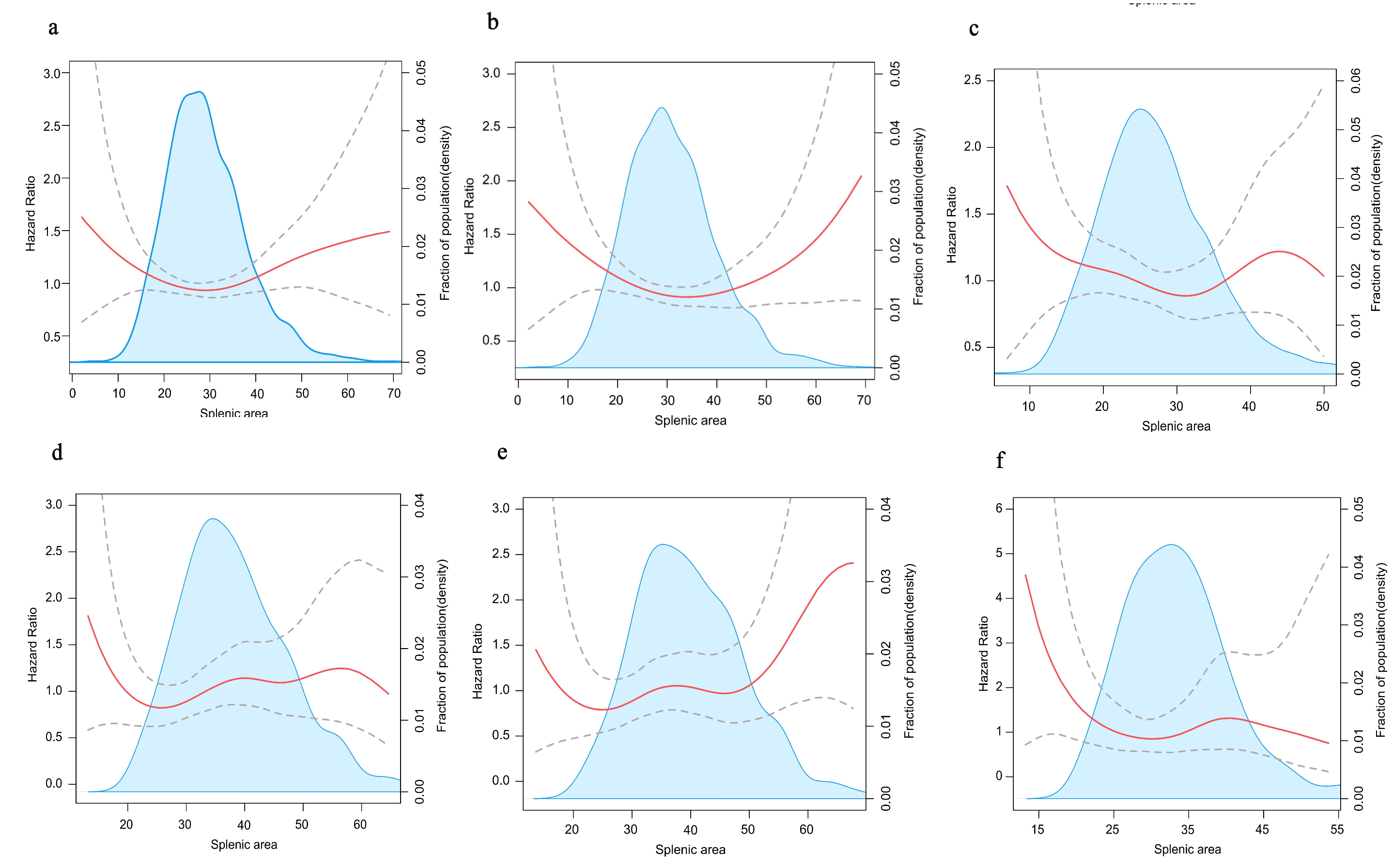

Supplement: Supplementary file 2 — Supplementary Material 2: Mortality according to splenic area. Unadjusted (red line) hazards for mortality according to splenic area (cm2). Dashed lines represent 95% confidence intervals. The blue lines represent the fraction of the population with different levels of splenic area; total population of the discovery cohort (a); male of the discovery cohort (b); female of the discovery cohort (c); total population of the validation cohort (d); male of the validation cohort (e); female of the validation cohort (f). [file 40644_2023_640_MOESM2_ESM.png]
