# Supplementary material for: Preoperative splenic area as a prognostic biomarker of early-stage non-small cell lung cancer
Source: Cancer Imaging. 2023 Dec 1;23:116. doi: 10.1186/s40644-023-00640-0 (PMC10691021; doi:10.1186/s40644-023-00640-0)
Supplement: Supplementary file 3 — Supplementary Material 3 [file 40644_2023_640_MOESM3_ESM.docx]

**Supplement Table 1 Threshold values significantly associated with abnormal splenic area**

| Population group | Quantile (%) | Cutoff value of splenic area (cm^2^) | |
| --- | --- | --- | --- |
|  |  | Discovery cohort | Validation cohort |
| **Male** |  |  |  |
| The inflection point (lowest point) of the curve | - | 33.90 | 25.00 |
| Lower section of the lowest point | ≤ 75 | ≤ 29.37 | ≤ 23.06 |
| Upper section of the lowest point | > 25 | > 36.05 | > 31.14 |
| **Female** |  |  |  |
| The inflection point (lowest point) of the curve | - | 31.19 | 30.17 |
| Lower section of the lowest point | ≤ 67 | ≤ 25.50 | ≤ 26.12 |
| Upper section of the lowest point | > 33 | > 33.85 | > 33.86 |

**Supplement Table 2 Characteristics of the cohort at baseline in the validation cohort**

| Variable | Total (n = 608)**^1^** | Splenic area | | *P* value**^2^** |
| --- | --- | --- | --- | --- |
|  |  | Normal (n = 169)**^1^** | Abnormal (n = 439)**^1^** |  |
| Age, years |  |  |  |  |
| Mean (SD) | 68.12 (9.99) | 68.29 (9.62) | 68.05 (10.14) | 0.80 |
| Median (IQR) | 68.77(62.00,75.89) | 68.11(61.33,76.49) | 68.96(62.32,75.46) | 0.98 |
| Sex, n (%) |  |  |  | 0.044 |
| Female | 205 (33.72) | 68 (40.24) | 137 (31.21) |  |
| Male | 403 (66.28) | 101 (59.76) | 302 (68.79) |  |
| Smoking history, n (%) |  |  |  | 0.57 |
| Never smoker | 47 (7.73) | 10 (5.92) | 37 (8.43) |  |
| Current or former smoker | 144 (23.68) | 40 (23.67) | 104 (23.69) |  |
| Unknown | 417 (68.59) | 119 (70.41) | 298 (67.88) |  |
| Tumor location, n (%) |  |  |  | 0.06 |
| Upper lobe | 111 (18.26) | 36 (21.30) | 75 (17.08) |  |
| Non–upper lobe | 80 (13.16) | 14 (8.28) | 66 (15.03) |  |
| Other | 417 (68.59) | 119 (70.41) | 298 (67.88) |  |
| Tumor differentiation, n (%) |  |  |  | 0.80 |
| Moderate-Well | 97 (15.95) | 27 (15.98) | 70 (15.95) |  |
| Poor-undifferentiated | 27 (4.44) | 6 (3.55) | 21 (4.78) |  |
| Other | 484 (79.61) | 136 (80.47) | 348 (79.27) |  |
| T stage, n (%) |  |  |  | 0.77 |
| Tis-T1 | 164 (27.02) | 42 (24.85) | 122 (27.85) |  |
| T2 | 201 (33.11) | 59 (34.91) | 142 (32.42) |  |
| T3 | 71 (11.70) | 19 (11.24) | 52 (11.87) |  |
| T4 | 120 (19.77) | 37 (21.89) | 83 (18.95) |  |
| Unknown | 52 (8.55) | 12 (7.10) | 40 (9.11) |  |
| N stage, n (%) |  |  |  | 0.93 |
| N0 | 280 (46.05) | 77 (45.56) | 203 (46.24) |  |
| N1 | 37 (6.09) | 12 (7.10) | 25 (5.69) |  |
| N2- N3 | 239 (39.31) | 66 (39.05) | 173 (39.41) |  |
| Unknown | 52 (8.55) | 14 (8.28) | 38 (8.66) |  |
| Histologic Stage, n (%) |  |  |  | 0.80 |
| 0-Ⅰ | 183 (30.10) | 53 (31.36) | 130 (29.61) |  |
| Ⅱ | 70 (11.51) | 17 (10.06) | 53 (12.07) |  |
| Ⅲ | 305 (50.16) | 87 (51.48) | 218 (49.66) |  |
| Unknown | 50 (8.22) | 12 (7.10) | 38 (8.66) |  |
| Histologic type, n (%) |  |  |  | 0.83 |
| Adenocarcinoma | 209 (34.38) | 57 (33.73) | 152 (34.62) |  |
| Squamous cell carcinoma | 180 (29.61) | 48 (28.40) | 132 (30.07) |  |
| Other | 219 (36.02) | 64 (37.87) | 155 (35.31) |  |
| Pleural invasion, n (%) |  |  |  | 0.93 |
| Yes | 36 (5.92) | 10 (5.92) | 26 (5.92) |  |
| No | 103 (16.94) | 27 (15.98) | 76 (17.31) |  |
| Unknown | 469 (77.14) | 132 (78.11) | 337 (76.77) |  |
| Adjuvant chemotherapy, n (%) |  |  |  | 0.77 |
| Yes | 41 (6.74) | 12 (7.10) | 29 (6.61) |  |
| No | 149 (24.51) | 38 (22.49) | 111 (25.28) |  |
| Unknown | 418 (68.75) | 119 (70.41) | 299 (68.11) |  |

**Note:**

**^1^** Data are median (IQR)/ Mean (SD) or n (%).

**^2^** *P*-value, using Wilcoxon Mann-Whitney test, chi-square test or exact Fisher test depending on whether the variable is continuous or categorical.

**Supplement Table 3 Comparison of clinical characteristics of discovery cohort and validation cohort**

| Variable | Total**^1^**  (n = 3140) | Discovery cohort **^1^**  (n = 2532) | Validation cohort **^1^**  (n = 608) | *P*-value**^2^** |
| --- | --- | --- | --- | --- |
| Age, years |  |  |  |  |
| Mean (SD) | 61.00 (53.00,68.00) | 59.00 (52.00,66.00) | 68.77 (62.00,75.89) | <0.001 |
| Median (IQR) | 60.56 (10.30) | 58.82 (9.56) | 68.12 (9.99) | <0.001 |
| Sex, n (%) |  |  |  |  |
| Female | 1363 (43.41) | 1158 (45.73) | 205 (33.72) | <0.001 |
| Male | 1777 (56.59) | 1374 (54.27) | 403 (66.28) |  |
| Smoking history, n (%) |  |  |  | 0.001 |
| Never smoker | 1166 (37.13) | 1022 (40.36) | 144 (23.68) |  |
| Current or former smoker | 1523 (48.50) | 1476 (58.29) | 47 (7.73) |  |
| Unknown | 451 (14.36) | 34 (1.34) | 417 (68.59) |  |
| Tumor location, n (%) |  |  |  | <0.001 |
| Upper lobe | 1388 (44.20) | 1277 (50.43) | 111 (18.26) |  |
| Non–upper lobe | 1328 (42.29) | 1248 (49.29) | 80 (13.16) |  |
| Other | 424 (13.50) | 7 (0.28) | 417 (68.59) |  |
| Tumor differentiation, n (%) |  |  |  | 0.008 |
| Moderate-Well | 385 (12.26) | 288 (11.37) | 97 (15.95) |  |
| Poor-undifferentiated | 153 (4.87) | 126 (4.98) | 27 (4.44) |  |
| Other | 2602 (82.87) | 2118 (83.65) | 484 (79.61) |  |
| T stage, n (%) |  |  |  | <0.001 |
| Tis-T1 | 1253 (39.90) | 1089 (43.01) | 164 (26.97) |  |
| T2 | 1163 (37.04) | 962 (37.99) | 201 (33.06) |  |
| T3 | 364 (11.59) | 293 (11.57) | 71 (11.68) |  |
| T4 | 304 (9.68) | 184 (7.27) | 120 (19.74) |  |
| Unknown | 56 (1.78) | 4 (0.16) | 52 (8.55) |  |
| N stage, n (%) |  |  |  | <0.001 |
| N0 | 1826 (58.15) | 1546 (61.06) | 280 (46.05) |  |
| N1 | 270 (8.60) | 233 (9.20) | 37 (6.09) |  |
| N2- N3 | 589 (18.76) | 350 (13.82) | 239 (39.31) |  |
| Unknown | 455 (14.49) | 403 (15.92) | 52 (8.55) |  |
| Histologic Stage, n (%) |  |  |  | <0.001 |
| 0-Ⅰ | 1399 (44.55) | 1216 (48.03) | 183 (30.10) |  |
| Ⅱ | 480 (15.29) | 410 (16.19) | 70 (11.51) |  |
| Ⅲ | 831 (26.46) | 526 (20.77) | 305 (50.16) |  |
| Unknown | 430 (13.69) | 380 (15.01) | 50 (8.22) |  |
| Histologic type, n (%) |  |  |  | <0.001 |
| Adenocarcinoma | 2170 (69.11) | 1961 (77.45) | 209 (34.38) |  |
| Squamous cell carcinoma | 634 (20.19) | 454 (17.93) | 180 (29.61) |  |
| Other | 336 (10.70) | 117 (4.62) | 219 (36.02) |  |
| Pleural invasion, n (%) |  |  |  | <0.001 |
| Yes | 512 (16.31) | 43 (1.70) | 469 (77.14) |  |
| No | 2167 (69.01) | 2064 (81.52) | 103 (16.94) |  |
| Unknown | 461 (14.68) | 425 (16.79) | 36 (5.92) |  |
| Adjuvant chemotherapy, n (%) |  |  |  | <0.001 |
| Yes | 659 (20.99) | 241 (9.52) | 418 (68.75) |  |
| No | 1224 (38.98) | 1075 (42.46) | 149 (24.51) |  |
| Unknown | 1257 (40.03) | 1216 (48.03) | 41 (6.74) |  |
| Splenic area |  |  |  |  |
| Mean (SD) | 29.83 (10.75) | 28.65 (10.26) | 34.78 (11.35) | <0.001 |
| Median (IQR) | 28.32 (22.69,35.08) | 27.37 (22.03,33.74) | 33.28 (26.78,41.25) | <0.001 |

**Note:**

**^1^** Data are median (IQR)/ Mean (SD) or n (%).

**^2^** *P*-value, using Wilcoxon Mann-Whitney test, chi-square test or exact Fisher test depending on whether the variable is continuous or categorical.
